# *Carex borealifujianica* (Cyperaceae), a new species of the core Carex clade from Fujian, southeastern China

**DOI:** 10.1371/journal.pone.0264419

**Published:** 2022-03-31

**Authors:** Yi-Fei Lu, Xiao-Feng Jin, Ming-Jian Yu

**Affiliations:** 1 College of Life Sciences, Zhejiang University, Hangzhou, Zhejiang, People’s Republic of China; 2 State Key Laboratory of Subtropical Silviculture/Zhejiang Provincial Key Laboratory of Forest Aromatic Plants-based Healthcare Funcations/School of Forestry and Bio-Technology, Zhejiang A&F University, Hangzhou, Zhejiang, People’s Republic of China; Nanjing Institute of Geology and Palaeontology, Chinese Academy of Sciences, CHINA

## Abstract

A new species, *Carex borealifujianica* Y.F. Lu & X.F. Jin (Cyperaceae, sect. *Occlusae* of core Carex clade) is described and illustrated from northern Fujian, China. In addition to morphological comparisons with its relatives, comparative micromorphology of utricles and achenes of seven species in *Carex* sect. *Occlusae* was examined. Micromorphology of utricles and achenes revealed the similarity of *Carex borealifujianica* and *C*. *ligulata*. Morphologically, this new species is similar to *Carex ligulata* in having lateral spikes remote and densely flowered, as well as utricles densely hispidulous, but differs in having 2 or 3 narrowly clavate staminate spikes, leaves 2.5–5 mm wide with sheaths sparsely pilose, and achenes emarginate at the apex. The phylogenetic analysis from two nuclear DNA regions (ETS and ITS) and two chloroplast DNA regions (*mat*K and *trn*L-F) of 68 taxa resolved *C*. *borealifujianica* as a distinct species.

## Introduction

*Carex* L., a cosmopolitan genus containing ca. 2000 known species divided into 130+ sections, is one of the largest genera of flowering plants [1–4]. The genus is distributed across every continent with the exception of Antarctica and grows in various habitats ranging from tropical forests to Arctic tundra [5]. The genus *Carex* is unique within the family Cyperaceae and characterized by its unisexual flowers with pistillate flowers surrounded by a fully or partially connate sac-like structure (utricle) [6].

The genus *Carex* (sedges) is also notoriously difficult in both taxonomy and phylogeny. In the past ten years, phylogenetic studies revealed that *Carex* as traditionally circumscribed is largely paraphyletic [6, 7]. The genus *Carex* now includes *Cymophyllus* Mack., *Kobresia* Willd., *Schoenoxiphium* Nees, and *Uncinia* Pers. Recent studies revealed *Carex* is comprised of six major clades corresponding to six subgenera: 1) the Siderosticta clade (subg. *Siderosticta* Waterway), sister to the rest of the genus and the earliest diverging lineage, with large chromosomes but low numbers of chromosomes; 2) the Schoenoxiphium clade (subg. *Psyllophorae* (Degl.) Peterm.), a relatively old group showing a striking disjunction of Western Palearctic, South America and Southern Africa; 3) the Unispicate clade (subg. *Euthyceras* Peterm.), with inflorescence usually reducing to a single spike or paniculiform, rarely racemose and spike-like; 4) the Uncinia clade (subg. *Uncinia* (Pers.) Peterm.), almost distributed in Western Hemisphere, with most species having solitary spike; 5) the Vignea clade (subg. *Vignea* (P. Beauv. ex T. Lestib.) Heer), including nearly all species traditionally placed in subg. *Vignea*; 6) the core Carex clade (subg. *Carex*), with the majority of subg. *Carex*, subg. *Vigneastra* and subg. *Phyllophora* of Kükenthal [8–13].

The species diversity within *Carex* in China is incredibly rich (including the former genus *Kobresia*) with 570+ species and 276 endemics [14]. Explorations after the publication of the *Flora of China* (Cyperaceae), have disclosed many new species of *Carex* following studies of regional floras, which shows *Carex* to be a mega-diverse genus in China [15–38]. Due to the morphological variability in *Carex*, as well as high species diversity, sectional assignment is a gateway to species identification. A section, as an infrageneric taxonomic category, is a very important taxonomic rank used widely in floras [11]. Section *Occlusae* is characterized by having hairy utricles, culm nodes exserted beyond the closed leaf sheaths, and initially included four species (*Carex ligulata* Nees, *C*. *maubertiana* Boott, *C*. *hebecarpa* C.A. Mey. and *C*. *sclerocarpa* Franch.). Now, it includes about eight species, of which seven occur in China, and are distributed in E or SE Asia [14, 34].

Mount Wuyi, located in northern Fujian and southern Jiangxi provinces, is a famous mountain in Southeast China and is one of the typical ‘Danxia’ landforms referring to various landscapes found in southeast, southwest and northwest China that consist of a red bed characterized by steep cliffs. Mount Wuyi is one of the biodiversity hotspots in China [39]. The flora of Mount Wuyi contains 3700+ vascular plants including a number of species named from this mountain, e.g. *Dryopteris wuyishanica* Ching & P.S. Chiu (Dryopteridaceae), *Berberis wuyiensis* C.M. Hu (Berberidaceae), *Sinosenecio wuyiensis* Y.L. Chen (Asteraceae), *Thalictrum wuyishanicum* W.T. Wang & S.H. Wang (Ranunculaceae), *Rhododendron wuyishanicum* L.K. Ling (Ericaceae), *Impatiens wuyiensis* J.S. Wang, Y.F. Lu & X.F. Jin (Balsaminaceae), *Carex wuyishanensis* S.Yun Liang (Cyperaceae), *Pleioblastus wuyishanensis* Q.F. Zheng & K.F. Huang and *Yushania wuyishanensis* Q.F. Zheng & K.F. Huang (Poaceae). Wuyishan National Nature Reserve and National Park attracts botanists and ecologists. In the present study, we recognize a recently collected sedge from Mount Wuyi as a new species. This new species is similar to *C*. *ligulata*, but differs in a number of morphological characters. We studied the morphology of the species, and determined its phylogenetic position, along with describing it below.

## Materials and methods

### Ethics statement

Twenty-two samples representing 18 taxa were used for molecular phylogenetic analyses without any protected species. The materials used for DNA extraction in this work were collected in Lin’an, Longquan, Pan’an counties of Zhejiang Province, Wuyishan City and Wuyishan Scenic Area of Fujian Province, and Maqu County of Gansu Province, all with permission of local government agencies or officers. Specimen examinations were permitted by curators of several herbaria, and the other sequences were directly downloaded from GenBank. A complete list of the samples used for phylogenetic analysis with GenBank accession numbers is shown in Table 1.

**Table 1:** Sampling information of the species used in the phylogenetic analysis of *Carex borealifujianica*.

Species	Locality	Voucher	GenBank accession number			
			ETS	ITS	matK	trnL-F
*Carex borealifujianica1*	China: Fujian, Wuyishan	*X*.*F*. *Jin 4176*	MW458982	MW459013	MW459080	MW459044
*Carex borealifujianica2*	China: Fujian, Wuyishan	*X*.*F*. *Jin 4159–1*	MW458985	MW459016	MW459083	MW459047
*Carex borealifujianica3*	China: Fujian, Wuyishan	*X*.*F*. *Jin 4159–2*	MW458986	MW459017	MW459084	MW459048
*Carex digitata*	Italy: Tuscany	*P*. *Jimenez-Mejias 27PJM10*	MN759970	MN762307	MN763754	-
*Carex ericetorum*	Italy: Abruzzo	*P*. *Jimenez-Mejias et al*. *239PJM10*	MN760026	MN762286	MN763731	-
*Carex halleriana*	Spain: Huelva	*E*. *S*. *Gullon et P*. *Weickert s*.*n*.	MN760532	MN762338	MN763694	-
*Carex hebecarpa*	China: Zhejiang, Lin’an	*X*.*F*. *Jin 4152*	MW458990	MW459021	MW459088	MW459052
*Carex lativena*	USA: New Mexico	*R*.*D*. *Worthington 30373*	MN760528	MN761490	GU173073	-
*Carex ligulata1*	China: Fujian, Wuyishan	*X*.*F*. *Jin 4162*	MK481321	MK481341	MT920810	MK481361
*Carex ligulata2*	China: Fujian, Wuyishan	*X*.*F*. *Jin 4170*	MW458987	MW459018	MW459085	MW459049
*Carex mabilliana*	France: Corsica	*J*.*M*. *Tison s*.*n*.	MN760494	MN762305	MN763686	-
*Carex maubertiana1*	China: Zhejiang, Pan’an	*X*.*F*. *Jin 3703*	MW458988	MW459019	MW459086	MW459050
*Carex maubertiana2*	China: Fujian, Wuyishan	*X*.*F*. *Jin 4175*	MW458989	MW459020	MW459087	MW459051
*Carex melanocarpa*	Russia	*S*. *Kharkevich et T*. *Buch 969e*	MN760025	-	MN763730	-
*Carex ornithopoda*	Montenegro	*P*. *Jimenez-Mejias et J*. *Pantović 187PJM10*	MN759995	MN762308	MN763753	-
*Carex pallens*	Finland: Uusimaa	*M*. *Piirainen et al*. *2219*	MN759997	MN762329	MN763411	-
*Carex phyllocephala*	China: Guangdong, Conghua	*S*.*H*. *Jin et al*. *CH09009*	MW458983	MW459014	MW459081	MW459045
*Carex planostachys*	USA: Texas	*R*.*F*.*C*. *Naci 10156*	MN760531	MN761486	GU173407	-
*Carex przewalskii*	China: Gansu, Maqu	*Y*.*F*. *Lu et X*. *Geng 3*	MZ516812	MZ516813	MZ516814	MZ516815
*Carex pseudophyllocephala*	China: Yunnan, Fugong	*L*. *Heng et al*. *20997*	-	MN761973	-	-
*Carex quadriflora*	Korea?	*anonymous*, *s*.*n*.	MN759998	MN762064	MN763044	-
*Carex rorulenta*	Spain: Balearic Islands	*H*. *Merxmüller et al*. *100/57*	MN760530	MN762336	MN763693	-

### Nomenclature

The electronic version of this article in Portable Document Format (PDF) in a work with an ISSN or ISBN will represent a published work according to the International Code of Nomenclature for algae, fungi, and plants; thus, the new names contained in the electronic publication of a PLOS ONE article are effectively published under that Code from the electronic edition alone, so there is no longer any need to provide printed copies. In addition, new names contained in this work have been submitted to IPNI, from where they will be made available to the Global Names Index. The IPNI LSIDs can be resolved and the associated information viewed through any standard web browser by appending the LSID contained in this publication to the prefix http://ipni.org/. The online version of this work is archived and available from the following digital repositories: PubMed Central, LOCKSS.

### Population sampling and specimen examination

During our explorations on Mount Wuyi in 2018 and 2019, we collected a *Carex* with densely hispidulous utricles. The plant was easily recognized as a member of *Carex* sect. *Occlusae* C.B. Clarke.

A total of ten populations were examined for morphological analysis, including one population of the new species (*Carex borealifujianica*), six populations of *C*. *ligulata*, and three populations of *C*. *hebecarpa*. The specimens are conserved in the following herbaria: Hangzhou Normal College (HTC), South China Botanical Garden (IBSC), Institute of Botany, Chinese Academy of Sciences (PE) and University of Tokyo (TI), and collection information is shown in Table 2.

**Table 2:** Collection information of ten populations used to morphological analysis.

Species/population name	Voucher (Herbarium)	Number of individuals	Locality
*Carex borealifujianica*/ FJ-B	*Y*.*F*. *Lu 177* (HTC)	3	Dawangfeng, Wuyishan, Fujiang
	*Y*.*F*. *Lu 178* (HTC)	2	Dawangfeng, Wuyishan, Fujiang
	*Y*.*F*. *Lu 179* (HTC)	4	Dawangfeng, Wuyishan, Fujiang
	*X*.*F*. *Jin & Y*.*F*. *Lu 4159* (HTC)	3	Dawangfeng, Wuyishan, Fujiang
	*X*.*F*. *Jin & Y*.*F*. *Lu 4160* (HTC)	3	Dawangfeng, Wuyishan, Fujiang
	*X*.*F*. *Jin & Y*.*F*. *Lu 4161* (HTC)	3	Dawangfeng, Wuyishan, Fujiang
	*X*.*F*. *Jin & Y*.*F*. *Lu 4176* (HTC)	4	Huiyansi, Wuyishan, Fujiang
*C*. *hebecarpa*/ SC-H	*S*. *Jiang 7035* (PE)	2	Beichuan, Sichuan
*C*. *hebecarpa*/ TI-H	*J*.*S Ying & D*.*Y*. *Hong 650623* (PE)	2	Zhimo, Yigong, Tibet
*C*. *hebecarpa*/ YN-H	*T*.*N*. *Liou 13541* (IBSC)	1	without precise locality, Yunnan
	*T*.*N*. *Liou 14801* (IBSC)	1	without precise locality, Yunnan
*C*. *ligulata*/ AH-L	*Y*.*F*. *Lu & B*.*Y*. *Ding 219* (HTC)	11	Tiantangzhai, Jinzhai, Anhui
*C*. *ligulata*/ FJ-L	*Y*.*F*. *Lu 180* (HTC)	3	Dawangfeng, Wuyishan, Fujiang
	*Y*.*F*. *Lu 181* (HTC)	2	Dawangfeng, Wuyishan, Fujiang
*C*. *ligulata*/ HN-L	*anonymous s*.*n*. (PE)	3	Anjiang, Hunan
	*Anjiang Agri*. *School 418* (PE)	2	1^st^ District, Anjiang, Hunana
	*Z*.*T*. *Li 798* (PE)	1	1^st^ District, Anjiang, Hunana
	*Z*.*T*. *Li 545* (PE)	2	1^st^ District, Anjiang, Hunana
*C*. *ligulata*/ JX-L	*X*.*X*. *Yang 830420* (IBSC)	1	Shaxi, Yongfeng, Jiangxi
	*M*.*X*. *Nie 8627* (IBSC)	1	Chongyi, Jiangxi
	*C*.*M*. *Tan s*.*n*. (HTC)	8	Lushan, Jiujiang, Jiangxi
*C*. *ligulata*/ SCCQ-L	*Ins*. *Biol*. *Exp*. *23695* (IBSC)	1	Moxi, Luding, Sichuan
	*T*.*L*. *Dai 104080* (IBSC)	1	Penjiashan, Chengkuo, Chongqing
	*K*.*C*. *Kuan et al*. *2045* (PE)	2	Jinfoshan, Nanchuan, Chongqing
	*K*.*C*. *Kuan et al*. *1266* (PE)	2	Jinfoshan, Nanchuan, Chongqing
	*anonymous 91934* (PE)	1	Jinfoshan, Nanchuan, Chongqing
	*K*.*L*. *Chü 1529* (PE)	1	Jinfoshan, Nanchuan, Chongqing
*C*. *ligulata*/ JA-L	*anonymous s*.*n*. (TI)	3	Takaoka-gun, Kochi-ken, Japan
	*T*. *Miyazaki 508091* (TI)	2	Takaoka-gun, Kochi-ken, Japan
	*H*. *Ohashi s*.*n*. (TI)	1	Nangoku-shi, Kochi-ken, Japan
	*S*. *Saito 816* (TI)	2	Suzaki-city, Kochi-ken, Japan
	*S*. *Saito s*.*n*. (TI)	1	Ohmi, Yamagu-chi, Japan
	*Mitani s*.*n*. (TI)	1	Sanuki-shi, Kagawa-ken, Japan

### Morphological observations and statistical analysis

Morphological terminology used in the description of the species mainly follows Dai et al. (2010) and Jiménez-Mejías et al. (2016) [11, 14]. Quantitative characters, including culm height, leaf width, number, length and width of staminate and pistillate spikes, length of peduncle of staminate spike, pistillate flowers per spike, length of pistillate scales, utricle and achenes, were measured on all the samples. The density of utricles was calculated by utricle number per spike length, and the other qualitative characters were directly obtained and assigned by examining specimens. The size measurements of utricles and achenes of each individual were randomly measured from a total of 20 utricles and achenes of different spikes. Eighty individuals and 17 diagnostic characters were analyzed, and all diagnostic characters are presented in Table 3. A data matrix using 17 characters was produced for each population with each individual as an operational taxonomic unit (OTU) (Table 3). SPSS version 11.5 software was used to analyze the variation of quantitative characters, and principal component analysis (PCA) was used to determine the similarities among populations based on 17 characters in RStudio [40] using the functions of “scale”, “princomp” and “predict”; a 95% confidence interval was employed using the function “stat_ellipse” in the “ggplot2” package.

**Table 3:** Variation of the diagnostic characters of the ten populations studied.

**Population name**	**Culm height (cm)**	**Indumentum on basal sheath**	**Leaf width (mm)**	**Number of staminate spikes**	**Length of staminate spikes (cm)**	**Width of staminate spikes (mm)**	**Number of pistillate spikes**	**Length of pistillate spikes (cm)**
FJ-B [22]	72.41±2.95	pilose (1) [22]	4.12±0.08	2.61±0.12	0.93±0.05	0.98±0.03	3.61±0.12	2.87±0.09
SC-H [2]	23.00±2.32	glabrous (0) [2]	3.97±0.09	1.00±0.00	2.23±0.21	0.99±0.05	3.29±0.18	3.03±0.29
TI-H [2]	63.17±2.46	glabrous [2]	5.04±0.31	1.00±0.00	1.93±0.09	0.73±0.04	4.00±0.00	2.91±0.15
YN-H [2]	34.00±1.00	glabrous [2]	3.47±0.19	1.00±0.00	1.48±0.06	0.78±0.03	3.71±0.18	1.98±0.11
AH-L [11]	59.25±3.27	glabrous [11]	9.77±0.24	1.00±0.00	1.31±0.07	1.15±0.11	5.36±0.15	2.35±0.13
FJ-L [5]	49.33±5.57	glabrous [5]	7.01±0.18	1.00±0.00	1.85±0.12	1.19±0.04	3.17±0.31	2.33±0.13
HN-L [8]	51.56±2.90	glabrous [8]	8.94±0.22	1.00±0.00	1.80±0.11	1.33±0.07	5.38±0.32	2.77±0.11
JX-L [10]	60.60±1.86	glabrous [10]	7.09±0.21	1.00±0.00	1.44±0.10	1.04±0.14	4.45±0.25	1.97±0.10
SCCQ-L [8]	62.38±3.62	glabrous [8]	9.00±0.26	1.00±0.00	1.62±0.21	1.25±0.13	6.00±0.42	3.24±0.14
JA-L [10]	45.68±2.20	glabrous [10]	7.68±0.11	1.00±0.00	1.88±0.13	1.35±0.07	5.05±0.17	2.66±0.10

**Population name**	**Width of pistillate spikes (mm)**	**Utricle density(cm)**	**Utricle length (mm)**	**Veins on utricles**	**Shape of pistillate scales**	**Length of pistillate scales (mm)**	**Indumentum on pistillate scales**	**Achene shape**	**Achene length (mm)**
FJ-B [22]	3.63±0.04	8.40±0.19	4.22±0.03	conspicuous (1) [22]	ovate (0) [22]	2.48±0.03	glabrous (0) [1], villose (1) [21]	ovoid (0) [2], obovoid (1) [20]	2.31±0.01
SC-H [2]	2.49±0.07	8.12±0.34	3.62±0.16	inconspicuous (0) [2]	broadly ovate (1) [1], ovate [1]	1.74±0.05	glabrous [2]	obovoid [1], ovoid [1]	2.22±0.03
TI-H [2]	2.24±0.38	5.69±0.27	3.77±0.03	inconspicuous [2]	broadly ovate [2]	1.76±0.05	glabrous [2]	ovoid [2]	2.39±0.04
YN-H [2]	2.20±0.11	6.36±0.22	3.31±0.05	inconspicuous [2]	ovate [2]	1.82±0.06	glabrous [2]	ovoid [2]	2.12±0.06
AH-L [11]	4.98±0.11	17.94±0.67	3.98±0.05	inconspicuous [11]	broadly ovate [1], ovate [10]	2.36±0.06	glabrous [11]	obovoid [11]	2.45±0.02
FJ-L [5]	4.18±0.13	11.90±0.62	4.28±0.04	inconspicuous [5]	broadly ovate [2], ovate [3]	2.29±0.06	glabrous [5]	obovoid [5]	2.52±0.03
HN-L [8]	5.10±0.09	19.23±0.78	4.22±0.07	inconspicuous [8]	broadly ovate [1], ovate [7]	2.67±0.06	glabrous [8]	obovoid [8]	2.57±0.07
JX-L [10]	4.79±0.10	17.58±0.52	3.97±0.03	inconspicuous [10]	broadly ovate [5], ovate [5]	2.37±0.06	glabrous [10]	ovoid [4], obovoid [6]	2.55±0.02
SCCQ-L [8]	5.17±0.09	14.51±1.03	4.40±0.05	inconspicuous [8]	broadly ovate [2], ovate [6]	2.67±0.05	glabrous [8]	ovoid [5], obovoid [3]	2.66±0.04
JA-L [10]	5.54±0.11	15.14±0.45	4.13±0.04	inconspicuous [10]	ovate [10]	2.72±0.05	glabrous [10]	obovoid [10]	2.42±0.03

The values of quantitative characters were measured from different specimens/individuals, and are shown as mean±SD. States for qualitative characters are displayed in brackets at the first mention of each state. Values in square brackets correspond to the number of measured individuals.

### SEM observations

Scanning electron microscope (SEM) observations were conducted to compare the detailed micromorphology of utricles and achenes. The utricles and achenes of *Carex borealifujianica* and the other six species of *Carex* sect. *Occlusae* examined were studied from specimens.

Mature utricles were cleaned in 50% ethanol for 2 hr, and air dried. The cleaned utricles were mounted on stubs using double-sided adhesive tape, and coated with a layer of gold [22]. Mature achenes were initially soaked in a solution of concentrated sulfuric acid and acetic anhydride (vol.: vol. = 1: 9) for 12–18 hr, then rinsed in acetic acid for 10 min and water for 5 min, respectively. These samples were placed in a bath-type ultrasonic cleaner for 30 min with 70% ethanol to remove the cuticle and outer periclinal wall of the epidermis [41]. After air drying, the achenes were mounted and coated in a gold layer. The coated utricles and achenes were observed and photographed using an XL-30E scanning electron microscope (Philips, The Netherlands).

### Taxon sampling, DNA extraction, PCR and sequencing

A total of 22 samples representing 18 taxa were used for molecular phylogenetic analyses based on the results of the recent global megaphylogeny of *Carex* [11, 12], which indicated sect. *Occlusae* was a member of core Carex clade and closely related to sects. *Hallerianae*, *Acrocystis* and *Clandestinae*; 48 sequences were newly generated in this study (including those of 2 species, *C*. *phyllocephala* and *C*. *maubertiana*, which were sequenced for the first time); the other sequences were directly downloaded from GenBank (see Table 1).

Total genomic DNA was extracted from leaves dried in silica gel using TIANGEN Plant Genomic DNA Kit (Tiangen Biotech, Beijing, China). Two nuclear DNA regions (ETS and ITS) and two chloroplast DNA regions (*mat*K and *trn*L-F) used for phylogenetic analysis, were amplified using a DNA Engine PCR machine (Bio-Rad, Hercules, CA, USA) in 25 μL reactions. The amplification reaction mixture was prepared using Golden DNA Polymerase following the manufacturer’s instructions (Tiangen Biotech) and the amplified primers followed Starr et al. [42] for ETS, White et al. [43] for ITS, Starr and Ford [44] for *mat*K and Taberlet et al. [45] for *trn*L-F. The PCR program began at 94°C for 4 min, followed by 37 cycles of 94°C for 30 s, 55°C (57°C for ITS) for 30 s and 72°C for 1 min, followed by a 72°C extension for 10 min. All PCR products were electrophoresed on 1% agarose gels to verify product size.

The PCR products were purified with an AxyPrep PCR Clean-up Kit (Axygen Biotechnology Co., Hangzhou, China) following the manufacturer’s instructions. Sequencing was carried out on an ABI 3730 automated sequencer (Applied Biosystems, Foster City, CA, USA).

### Molecular phylogenetic analysis

Sequences were aligned using the online version of MAFFT [46], and then manually adjusted by MEGA X [47]. The ETS, ITS, *trn*L-F and *mat*K were combined in SequenceMatrix [48]. Optimal DNA substitute models for each region were selected using Akaike information criterion [49] in jModeltest v.2.1.3 [50], and resulted in GTR+G for ETS, ITS and *trn*L-F, GTR+I+G for *mat*K. Phylogenetic analysis was conducted in RAxML v. 8 [51] for Maximum Likelihood (ML) and Mrbayes v. 3.2.6 for Bayesian Inference (BI) [52]. For the ML analysis, a bootstrap analysis with 1000 replicates was conducted with a GTR+G model using other parameters by default. For the Bayesian analysis, four Metropolis-coupled Markov-chains Monte Carlo were run for 10^7^ generations with sampling every 1000 generations and until the standard deviation of split frequencies fell below 0.01. The first 2500 recovered trees were discarded (burn in = 0.25). Maximum parsimony bootstrap (BP) and Bayesian posterior probability (PP) were respectively calculated.

### Assessment of conservation status

International Union for Conservation of Nature (IUCN) Red List Categories and Criteria in 2012 was used to assess the conservation status of the new species. We followed the definitions and categories and used the field investigation data to estimate, such as population size, geographic range, and quality of habitat.

## Results

### Morphological variation within and among populations

**Fig 1:**
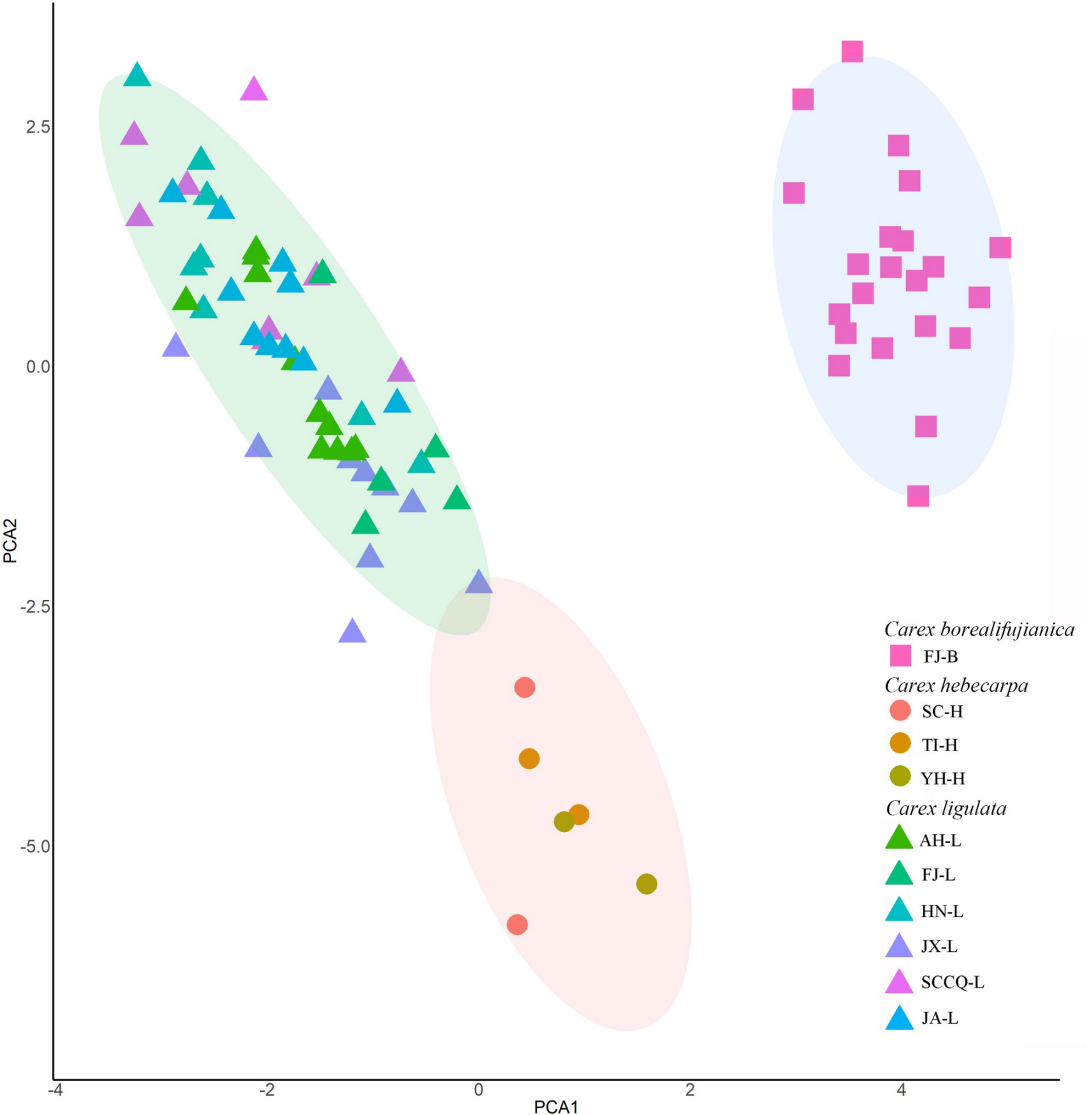
Principal component analysis of ten populations based on analysis of 17 diagnostic characters from 80 individuals. Populations names and detailed information follow Table 1.

The morphological characters we observed and measured are shown in Table 3 (see S1 Data), which indicates that most of the quantitative characters in the analysis are variable both within and across populations. In the PCA analysis, four principal components with eigenvalues >1 were extracted; these explained 39.61%, 19.78%, 11.85% and 6.09% of the variation among individuals, respectively. The first principal component had the highest loadings for leaf sheath indumentum, whether or not utricles had distinct thin veins, leaf width, indumentum of pistillate scales, number of staminate spikes, and density of utricles. The second principal component had the highest loadings for pistillate scale, utricle, and pistillate spike lengths. Based on the PCA of the diagnostic morphological characters, three groups were clearly recognized. The population named FJ-B was isolated from the other nine populations, whereas three populations (SC-H, TI-H and YN-H) formed the second group, close to the third group which included five populations from China (AH-L, FJ-L, HN-L, JX-L, and SCCQ-L) and one from Japan (JA-L). These three groups were identified with the three species *Carex borealifujianica*, *C*. *hebecarpa*, and *C*. *ligulata* (Fig 1).

One way analysis of variance (ANOVA) was performed among these three groups. Leaf widths of *Carex borealifujianica* and *C*. *hebecarpa* were significantly narrower than that of *C*. *ligulata* (4.06±0.12 mm and 4.11±0.30 mm vs. 8.30±0.21 mm, respectively). Utricle densities of *C*. *borealifujianica* and *C*. *hebecarpa* were significantly sparser than that of *C*. *ligulata* (8.45±0.29 /cm and 6.52±0.36 /cm vs. 16.44±0.50 /cm, respectively). Utricle lengths of *C*. *borealifujianica* and *C*. *ligulata* were longer than that of *C*. *hebecarpa* (4.23±0.04 mm and 4.15±0.04 mm vs. 3.46±0.10 mm, respectively). In addition, pistillate scale lengths of *C*. *borealifujianica* and *C*. *ligulata* were longer than that of *C*. *hebecarpa* (2.49±0.04 mm and 2.49±0.04 mm vs. 1.77±0.03 mm, respectively).

Some characters of *Carex borealifujianica* distinguished it from both *C*. *hebecarpa* and *C*. *ligulata*, such as indumentum on basal sheaths (pilose vs. glabrous), number of staminate spikes (2 or 3 vs. solitary, respectively), and veins on utricles (conspicuous vs. inconspicuous). From the above-mentioned analysis, the diagnostic characters distinguishing the new species from *Carex hebecarpa* and *C*. *ligulata* are shown in Table 4.

**Table 4:** Diagnostic characters distinguishing *Carex borealifujianica* from *C*. *hebecarpa* and *C*. *ligulate*.

Character	*C*. *borealifujianica*	*C*. *hebecarpa*	*C*. *ligulata*
Leaf			
width (mm)	2.6–5.0	3.2–5.4	5.7–11.7
sheath indumentum	pilose	glabrous	glabrous
Number of staminate spikes	2 or 3	1	1
Pistillate scale			
length (mm)	2.3–2.8	1.7–1.9	1.9–3.2
indumentum	villose	glabrous	glabrous
Utricle			
density (/cm)	5.5–10.8	5.4–8.1	8.6–22.6
length (mm)	3.8–4.6	3.2–3.8	3.4–5.0
veins on utricle	distinct	indistinct	indistinct

### Micromorphology of achenes and utricles

**Fig 2:**
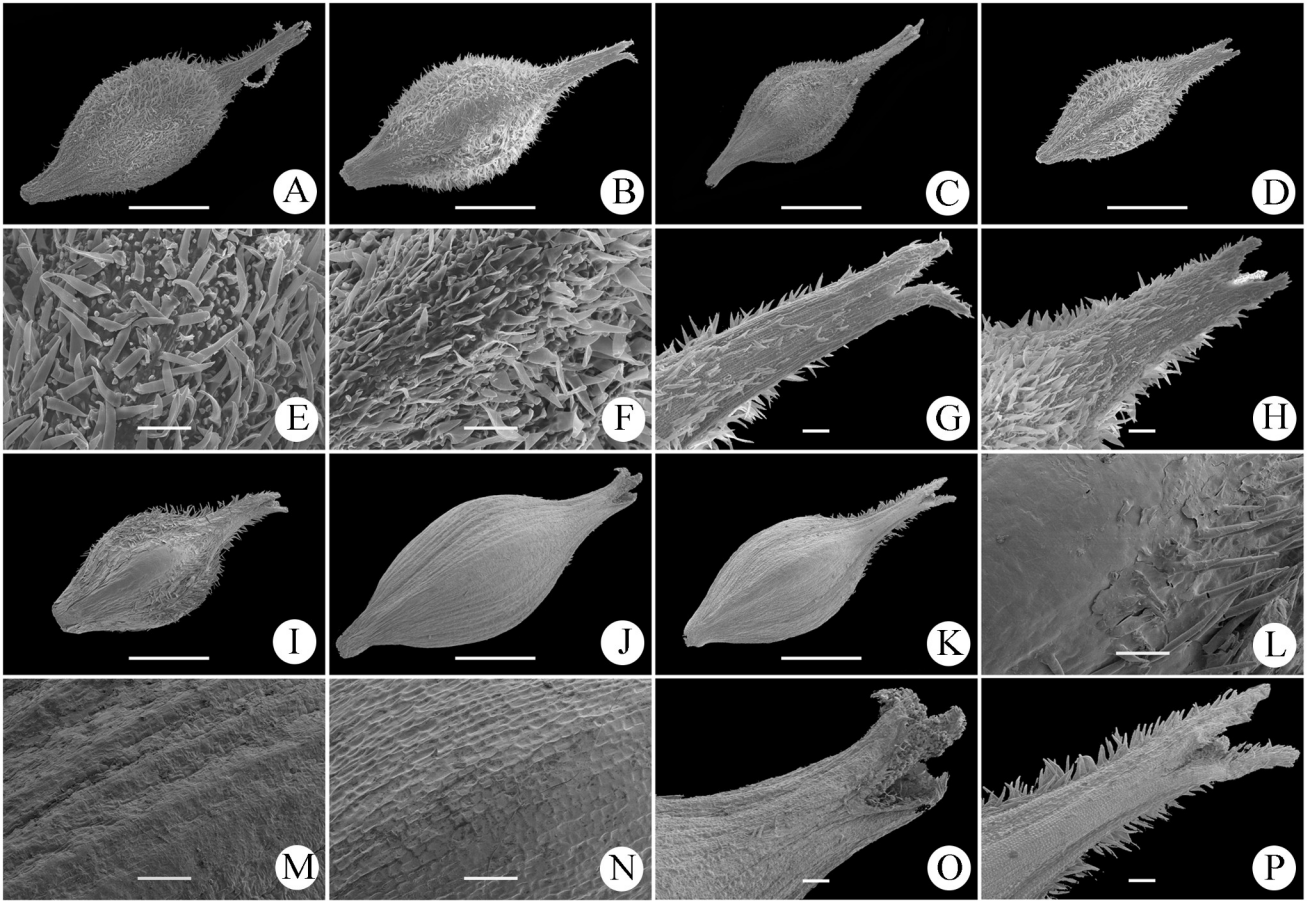
Scanning electron micrographs of the utricle micromorphology of seven species in *Carex* sect. *Occlusae*. A, E, *Carex borealifujianica*; B, G, *C*. *ligulata*; C. *C*. *hebecarpa*; D, F, H, *C*. *maubertiana*; I, L, *C*. *polycephala*; J, M, O, *C*. *poculisquama*; K, N, P, *C*. *pseudoligulata*. A–D, I–K, overview and shape (scale bar = 1 mm); E, F, L–N, surface indumentum (scale bar = 100 μm); G, H, O, P, beak (scale bar = 100 μm).

Utricles of all seven species in *Carex* sect. *Occlusae* were obovoid (excluding beak), and obtusely trigonous. Five species, *C*. *ligulata*, *C*. *hebecarpa*, *C*. *maubertiana*, *C*. *polycephala* and *C*. *borealifujianica* have densely hispidulous utricles, whereas the utricles of *C*. *poculisquama* were nearly glabrous and those of *C*. *pseudoligulata* were glabrous but densely hispidulous on the beak margin. *Carex borealifujianica* has utricles similar to those of *C*. *ligulata* in micromorphology (Fig 2).

**Fig 3:**
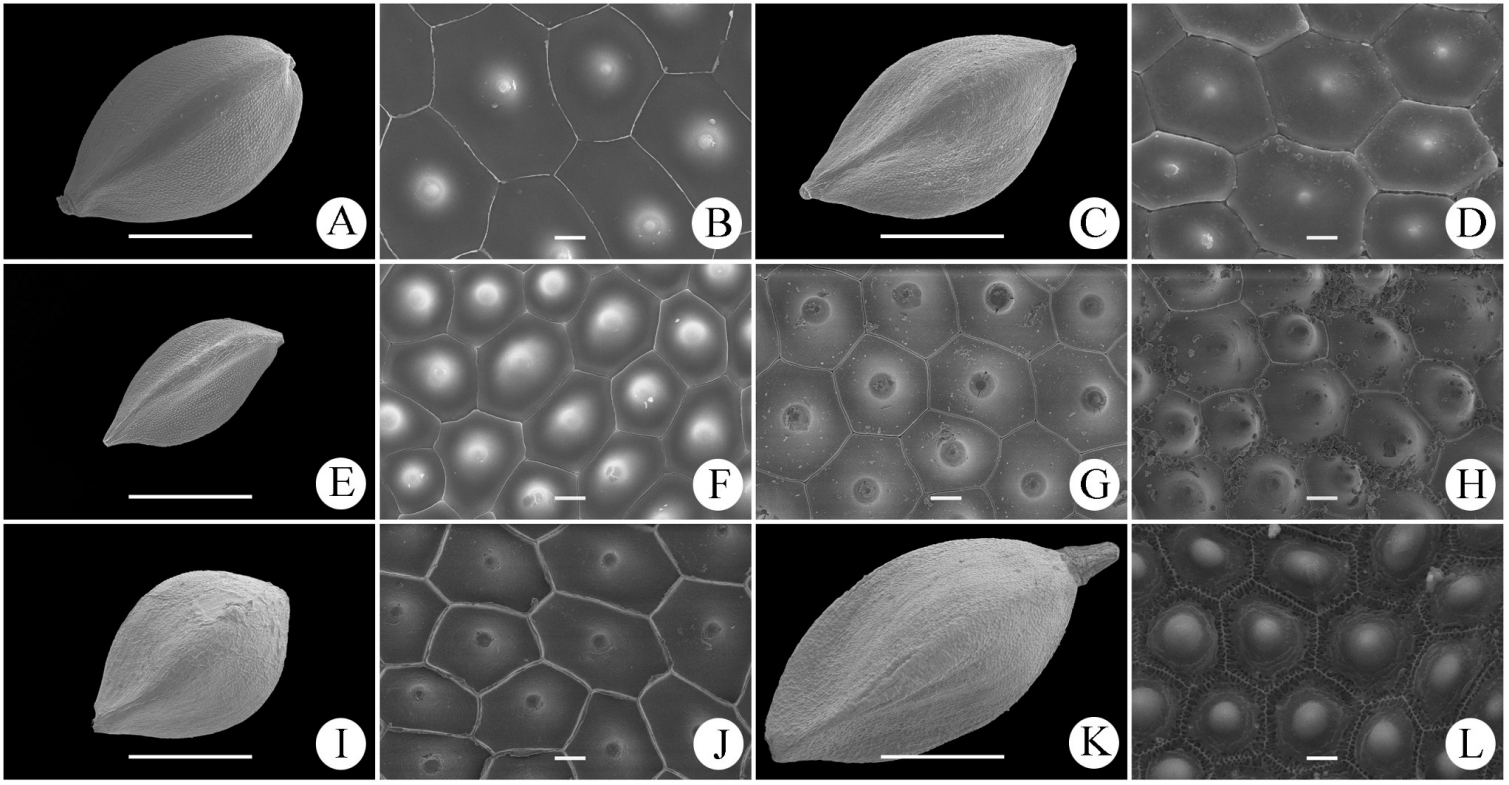
Scanning electron micrographs of the achene micromorphology of seven species in *Carex* sect. *Occlusae*. A, B, *Carex borealifujianica*; C, D, *C*. *ligulata*; E, F, *C*. *maubertiana*; G, *C*. *hebecarpa*; H, *C*. *pseudoligulata*; I, J, *C*. *polycephala*; K, L, *C*. *poculisquama*. A, C, E, I, K, overview and shape (scale bar = 1 mm); B, D, F–H, J, L, epidermal cell shape (scale bar = 10 μm).

Achene shape and surface sculpturing of *C*. *borealifujianica* and other six species in sect. *Occlusae* are shown in Fig 3. The trigonous achenes of most species were broadly obovoid, while those of *C*. *maubertiana* were obovoid. Moreover, only *C*. *borealifujianica* has achenes emarginate at the apex. The epidermal cells of all samples were irregularly 5–7-gonal, with straight inner anticlinal walls and a single silica body in each cell. *Carex borealifujianica* has achenes most similar to *C*. *ligulata* in micromorphology (Fig 3).

### Phylogenetic relationships

**Fig 4:**
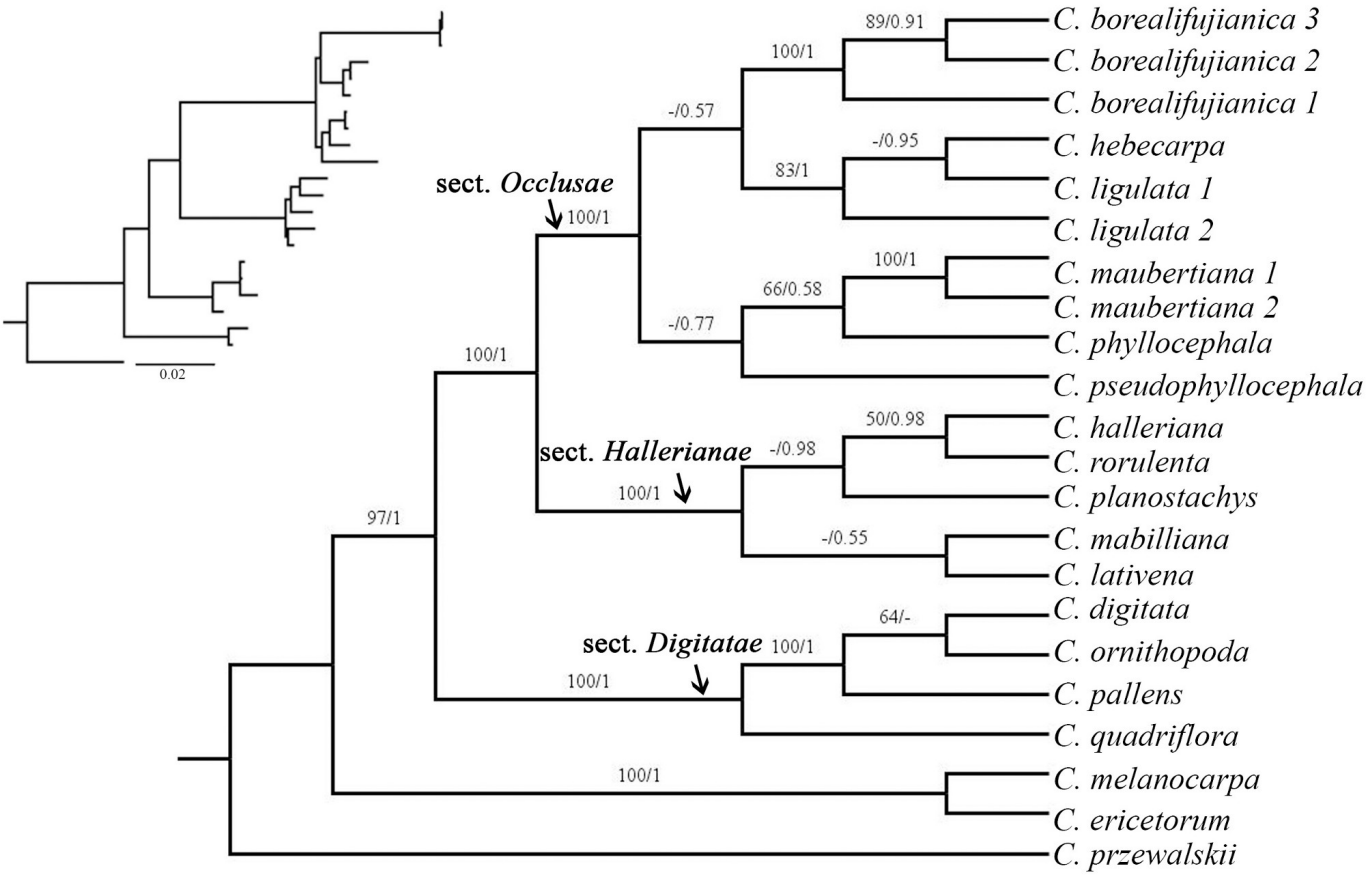
Maximum likelihood (ML) tree from the combined data (ETS, ITS, *mat*K and *trn*L-F), showing the relationship of *Carex borealifujianica*. Values above branches represent bootstrap values (BS) for maximum likelihood and Bayesian posterior probabilities (PP) respectively, and (-) indicates the BS<50% or PP<0.5.

The combined dataset contained 3057 bp aligned characters with the ETS, ITS, *mat*K and *trn*L-F containing 616 bp, 628 bp, 776 bp and 1037 bp, respectively. The topologies of BI and ML trees were generally congruent and recovered a similar phylogeny to those of previous studies [11, 12] (Fig 4). Six species in *Carex* sect. *Occlusae* clustered together and formed a distinct clade with high support (PP = 1, BS = 100%), which indicated this section is monophyletic. The three individuals of *Carex borealifujianica* formed a well-supported clade (PP = 1, BS = 100%). *Carex ligulata* is paraphyletic in a lineage that includes *C*. *hebecarpa*, indicating that further studies are required to clarify the relationship between these species.

This study indicated that *Carex* sect. *Occlusae* is monophyletic and recovered sister group to sect. *Hallerianae* (Aschers. & Graebn.) Rouy, which is consistent with previous studies [3, 12]. The results can be more reliable than the findings of previous studies since the additional sampling of sect. *Occlusae* compared with only two samples previously.

## Conclusions

Our study has found the population FJ-B from Fujian in SE China differs from *Carex ligulata* in several morphological characters and achene micromorphology. It was also found to display a distinct phylogenetic placement. Accordingly, we described it here as a new species.

**Fig 5:**
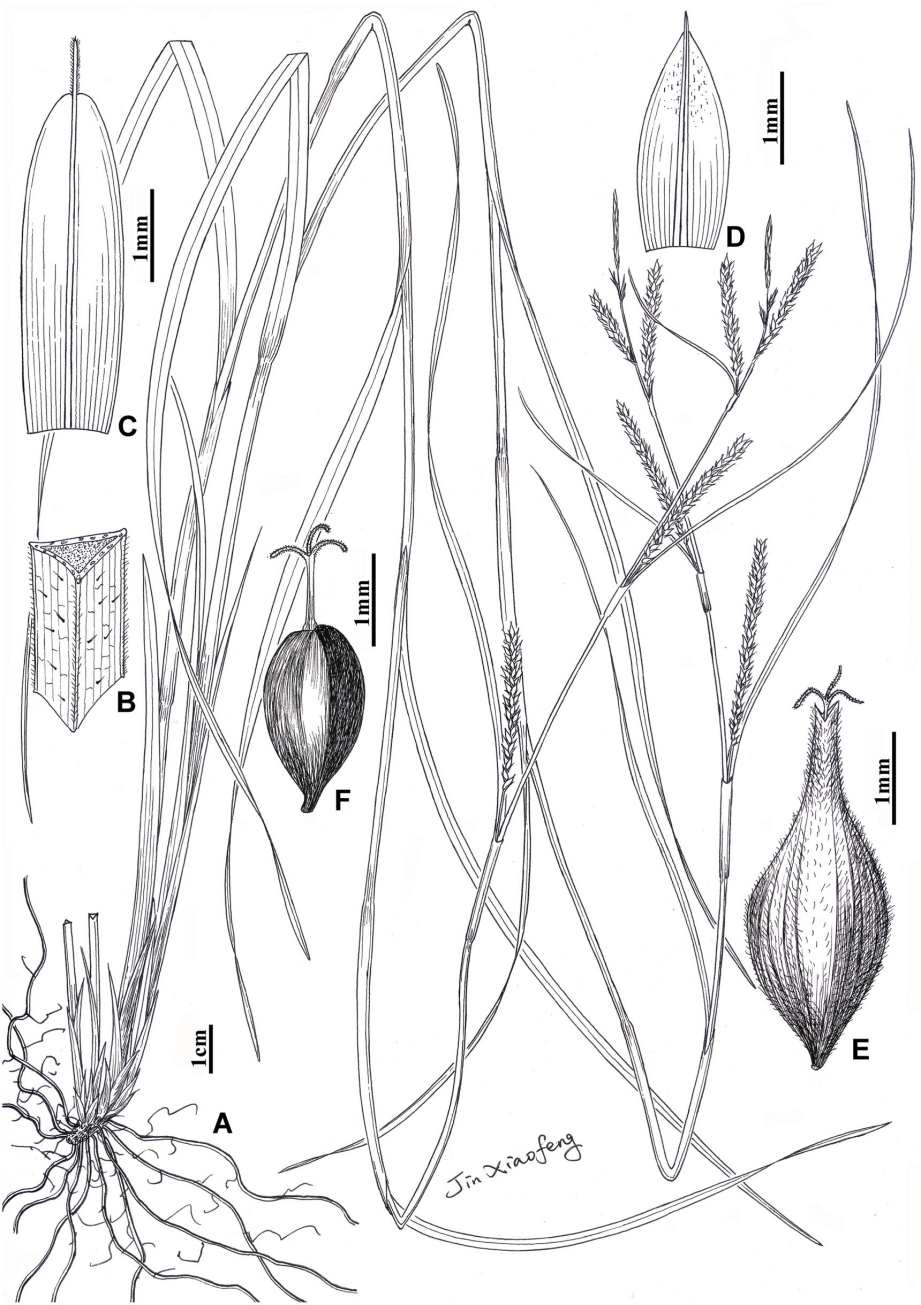
*Carex borealifujianica*. A, habit; B, indumentum on leaf sheath; C, staminate scale; D, pistillate scale; E, utricle; F, achene.

**Carex borealifujianica** Y.F. Lu & X.F. Jin, sp. nov. [urn:lsid:ipni.org:names: 77254902–1]. (Figs 2 and 5)

**Type**. CHINA. Fujian Province: Wuyishan City, Mount Wuyi, Shuiliandong, near Huiyuan Temple, roadside under forest, alt. 320 m, 27°40′38.18″N, 117°57′14.32″E 24 May 2018, X.F. Jin & Y.F. Lu 4176 (holotype: ZM; isotypes: HTC, PE, ZM).

*Haec species nova C. ligulatae Nees affinis est, sed foliis 2.5–5 mm latis, vaginis foliis sparse pilosis, spicis staminatis 2 vel 3, anguste clavatis, acheniis apice emarginatis differt*.

Herbaceous perennial. Rhizome woody, creeping or obliquely ascending, with many brown, fibrous adventitious roots. Culms loosely caespitose, 35–80 cm tall, acutely trigonous, slender to thick, upper parts scabrous, base with pale brown to dark brown bladeless sheaths. Leaves cauline, shorter than culms, 2.5–5 mm wide, flat, scabrous on both surfaces; sheaths not overlapping, 5–10 cm long, sparsely pubescent on the surface, but densely on the angles. Bracts leaf-like; lowermost 2 or 3 bracts shorter than inflorescence, with a 7–23 mm long sheathing base; upper ones longer or equaling the inflorescence, with a 3–5 mm long sheathing base or sheathless. Spikes 4–8, sparse in the base of the inflorescence, aggregated in the apex of the inflorescence; terminal staminate spikes 3 (rarely 2), narrowly clavate, 4–15 mm long, 1–1.5 mm wide; others pistillate, cylindrical, 1–3.6 cm long, 3.8–4.2 mm wide, ± densely flowered; lower peduncles extending to the apex of the bract sheaths, the upper ones enclosed. Staminate scales elliptic, yellowish brown, 4.5–5.5 mm long, apex obtuse, with yellow 1-veined costa excurrent into a 0.5–0.7 mm long scabrous awn. Pistillate scales ovate, pale brown to pale yellow, 2.5–2.8 mm long, apex mucronate, with green 3-veined costa. Utricles nearly erect or slightly patent, obovoid, obtusely trigonous, pale brownish green, longer than pistillate scales, 3.8–4.2 mm long, densely whitish hispidulous, lower part with 7 or 8 veins, base attenuate and short-stipitate, apex abruptly contracted into a 1–1.5 mm long erect beak, orifice with 2 short teeth. Achenes tightly enveloped by utricles, obovoid, trigonous, brown, 1.8–2.2 mm long, base short-stipitate, apex emarginate; style erect, base slightly thickened; stigmas 3.

**Etymology**. The specific epithet ‘borealifujianica’ refers to the type locality of this new species, Mount Wuyi, located in North Fujian Province.

**Phenology**. Flowering and fruiting from early May to mid-June.

**Fig 6:**
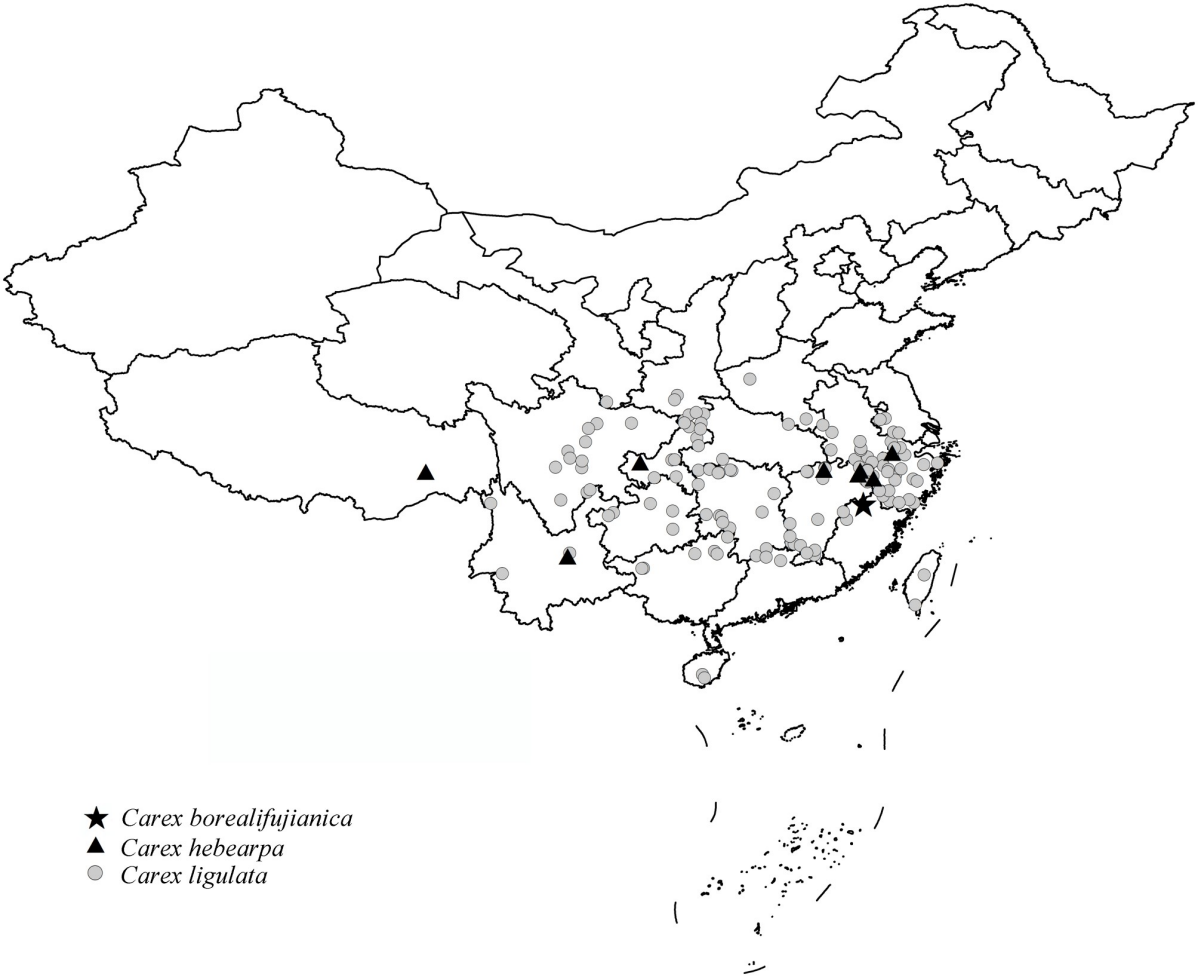
Distribution map of *Carex borealifujianica*, *C*. *hebecarpa* and *C*. *ligulata*. (Map source: https://www.webmap.cn/main.do?method=index; the distribution information is from collection notes of the specimens stored in ANUB, CDBI, HHBG, HNWP, HTC, IBK, KUN, LBG, NAS, PE, SZ, WUK and ZM).

**Distribution and habitat**. *Carex borealifujianica* is known only from the type locality (Fig 6). It grows on slopes, under forests or by streams at 220–320 m a.s.l.

**Additional specimens examined**. China. Fujian Province: Wuyishan City, Mount Wuyishan, Dawangfeng, on slope, alt. 220 m, 27°40′56.23″’N, 117°58′32.05″E, 23 May 2018, X.F. Jin & Y.F. Lu 4159 (HTC, ZM), 4160 (KUN, PE, ZM), 4161 (KUN, PE, ZM); the same locality, under forest, alt. 247 m, Y.F. Lu 177 (KUN, ZM), 178 (HTC, KUN, ZM), 179 (KUN, PE, ZM).

**Conservation status**: Vulnerable, VU B2ab(iii) (IUCN 2012). The occupancy of *Carex borealifujianica* is known to be less than 2000 km^2^, and it is distributed in no more than 10 locations. The quality of the habitat is continuously declining due to the disturbance caused by local tea plantation and tourism [53].

**Key to the species in *Carex* sect. *Occlusae* from China (mainly modified from Flora of China** [14]).

1a. Spikes approximate, usually aggregated at top of culm, subcapitately disposed; bracts aggregated.
  2a. Culms 12–15 cm tall; leaf blade 2–5 mm wide; utricles 2.3–2.5 mm long, ciliate on margins of beak…………………………….……………………….1. *C*. *pseudophyllocephala* L.K. Dai
  2b. Culms 20–60 cm tall; leaf blade 8–15 mm wide; utricles 2.8–3.5 mm long, densely hispidulous……………………………………………….….2. *C*. *phyllocephala* T. Koyama
1b. Spikes remote, racemosely disposed; bracts sparsely disposed.
  3a. Pistillate scales connate at base; pistillate spikes with sparse utricles; utricles subrhombic, trigonous, 5–5.3 mm long………………………………………………3. *C*. *poculisquama* Kük.
  3b. Pistillate scales not connate; pistillate spikes with dense utricles; utricles ovoid or obovoid, obtusely trigonous, 3–4.8 mm long.
    4a. Leaf blade 2–4 mm wide; pistillate spikes 3–4 mm wide; utricles sparsely disposed, subdistichous.
      5a. Staminate spikes 4–7.5 cm long; staminate scales oblanceolate, 7–8.5 mm long; utricles 3–3.6 mm long, glabrous…………………………………..4. *C*. *nodosa* S.R. Zhang et al.
      5b. Staminate spikes 1.5–2.5 cm long; staminate scales broadly obovate, 1.5–2 mm long; utricles 3–3.2 mm long, densely hispidulous……………………5. *C*. *hebecarpa* C.A. May.
    4b. Leaf blade 2.5–15 mm wide; pistillate spikes 3.8–6 mm wide; utricles densely disposed, multiseriate.
      6a. Utricles 3–5 mm long, densely hispidulous.
        7a. Leaf sheaths not overlapping, sheaths rather loosely enveloping culms; utricles 3.8–5 mm long.
          8a. Leaf blade 2.5–5 mm wide, sheaths sparsely pilose; staminate spikes 2 or 3, clavate; achenes emarginate at apex………5. ***C*. *borealifujianica*** Y.F. Lu & X.F. Jin
          8b. Leaf blade 5–15 mm wide, sheaths glabrous; staminate spike solitary, cylindrical; achenes acute at apex…………………………………………6. *C*. *ligulata* Nees
        7b. Leaf sheaths overlapping, sheaths tightly enveloping culms; utricles 3–3.2 mm long…………………………………………………….….7. *C*. *maubertiana* Boott
      6b. Utricles 3–3.5 mm long, hispidulous on upper margins and veins….….9. *C*. *pseudoligulata* L.K. Dai

## Supporting information

S1 Data The original data of the observed and measured morphological characters for the principal component analysis. (CSV)
